# S-layer associated proteins contribute to the adhesive and immunomodulatory properties of *Lactobacillus acidophilus* NCFM

**DOI:** 10.1186/s12866-020-01908-2

**Published:** 2020-08-12

**Authors:** Courtney Klotz, Yong Jun Goh, Sarah O’Flaherty, Rodolphe Barrangou

**Affiliations:** 1 Genomic Sciences Graduate Program North Carolina State University, Raleigh, NC USA; 2 Department of Food, Bioprocessing & Nutrition Sciences, North Carolina State University, Raleigh, NC USA

**Keywords:** *Lactobacillus*, S-layer, Probiotics, Cell surface

## Abstract

**Background:**

Surface layers (S-layers) are two-dimensional crystalline arrays of repeating proteinaceous subunits that form the outermost layer of many bacterial cell envelopes. Within the *Lactobacillus* genus, S-layer presence is frequently associated with probiotic-relevant properties such as improved adherence to host epithelial cells and modulation of the immune response. However, recent studies have demonstrated that certain S-layer functions may be supplemented by a novel subset of proteins embedded within its lattice, termed S-layer associated proteins (SLAPs). In the following study, four *Lactobacillus acidophilus* NCFM SLAPs (LBA0046, LBA0864, LBA1426, and LBA1539) were selected for in silico and phenotypic assessment.

**Results:**

Despite lacking any sequence similarity or catalytic domains that may indicate function, the genes encoding the four proteins of interest were shown to be unique to S-layer-forming, host-adapted lactobacilli species. Likewise, their corresponding deletion mutants exhibited broad, host-relevant phenotypes including decreased inflammatory profiles and reduced adherence to Caco-2 intestinal cells, extracellular matrices, and mucin in vitro.

**Conclusions:**

Overall, the data presented in this study collectively links several previously uncharacterized extracellular proteins to roles in the underlying host adaptive mechanisms of *L. acidophilus*.

## Background

*Lactobacillus* are Gram-positive, non-sporulating, anaerobic or microaerophilic bacteria, with complex nutritional requirements [1]. These versatile microorganisms inhabit diverse environments including dairy, meat and plant fermentations, as well as the gastrointestinal and urogenital tracts of humans and animals [2]. Although renowned for their applications in food and feed fermentations, several *Lactobacillus* species are also touted for their health-promoting, probiotic properties [3]. Probiotics are defined as “live microorganisms which when administered in adequate amounts confer a health benefit on the host” [4]. Efficacy tends to correlate with underlying host adaptive mechanisms such as bile and acid tolerance [5, 6], adhesion to mucus and epithelial cells [7, 8], and modulation of the immune response [9, 10] -- characteristics that can oftentimes be linked to the presence of specific extracellular proteins [2, 11, 12].

In general, *Lactobacillus* exoproteomes are composed of two main groups: secreted proteins that are released from the cell and surface-associated proteins, such as surface (S-)layers [2]. S-layers are two-dimensional crystalline arrays composed of numerous repeating subunits, S-layer proteins (Slps), that are inherently driven to self-assemble on the cell exterior [13, 14]. S-layers have been detected on several *Lactobacillus* species [1] and shown to play critical roles in adherence to intestinal cells [7, 15, 16] and host immunomodulation [10, 17]. However, recent studies have demonstrated the supplementation of these functions by various proteins embedded within the S-layer lattice. S-layer associated proteins, or SLAPs, first classified in industry-relevant strain *Lactobacillus acidophilus* NCFM [18], have since been identified on the surfaces of several species within the *L. acidophilus* homology group including *Lactobacillus helveticus*, *Lactobacillus crispatus*, *Lactobacillus amylovorus*, and *Lactobacillus gallinarum* [19]. To date, characterized SLAPs have exhibited a broad range of functions encompassing roles in cell maintenance processes such as cell division [20] and autolysin activity [21], as well as host adhesion [22] and immunomodulation [18, 23] mechanisms. Historically, selection of SLAP targets for deletion characterization was predominately based on their intriguing domain architecture or predicted function, but what about the extensive catalog of remaining proteins?

In the present study, four putative uncharacterized *L. acidophilus* SLAPs, routinely isolated from the cell surface [18, 19] and shown to exhibit either high relative abundance (LBA0864 and LBA1426) or significant induction in stationary growth phase (LBA0046 and LBA1539) [24], were selected for in silico and phenotypic analyses. Remarkably, despite having no sequence homology or predicted catalytic domains, all four genes were detected only in S-layer-forming, host-adapted lactobacilli species. Likewise, their corresponding deletion mutants demonstrated host-relevant phenotypes including modified immunogenicity profiles and reduced adherence to Caco-2 intestinal cells, extracellular matrices (ECMs) and mucin in vitro. Collectively, these results suggest that our four SLAPs of interest contribute to the survival and persistence *L. acidophilus* within the confines of the host gastrointestinal tract.

## Results

### Genetic analysis of SLAP loci

**Fig. 1:**
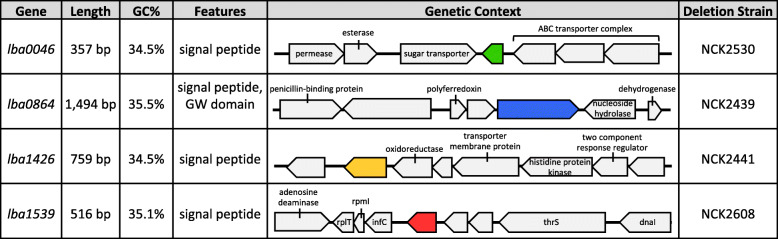
Schematic representation of the four S-layer associated proteins of interest. Table includes the gene length, GC percentage, interesting features, and genetic context of the four S-layer associated protein targets as well as their corresponding deletion strains. Genes are colored as follows, *lba0046* green, *lba0864* blue, *lba1426* yellow, and *lba1539* red. Unlabeled gray arrows represent genes of unknown function

Selected genes were shown to encode four previously uncharacterized surface proteins with no predicted function or COG. Their genetic layouts were analyzed on both a nucleotide and amino acid level (Fig. 1). Gene sizes ranged from 357 to 1494 bp, exhibited low GC content (34.5–35.5%), and were distributed randomly throughout the *L. acidophilus* chromosome. Though each possessed an N-terminal signal sequence, indicative of secretion and/or incorporation into cell wall/cell membrane components, only *lba0864* contained a GW (Gly-Tryp) dipeptide surface anchor. Genetic context was used to gain insight into the potential function of these proteins. The smallest of the genes analyzed, *lba0046*, was flanked by two transporters, namely a sugar transporter and an ABC transporter of unknown substrate specificity. The largest gene, *lba0864*, was located upstream of a nucleoside hydrolase and downstream of a hypothetical protein and polyferredoxin gene. The locus encoding LBA1539 was bordered by a 50S ribosomal protein complex and two hypothetical proteins, while *lba1426* was situated upstream of a two-component regulatory system. None of the analyzed genes were predicted to be part of an operon.

### Mapping target SLAPs to a phylogenetic tree

**Fig. 2:**
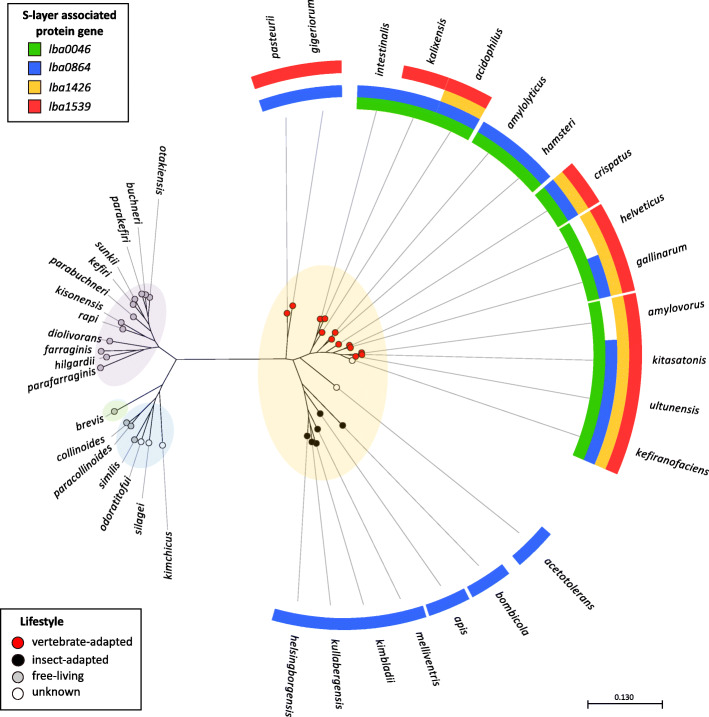
Presence/absence of *lba0046*, *lba0864*, *lba1426*, and *lba1539* mapped to a phylogenetic tree. Gene targets were mapped to a phylogenetic tree constructed based on the pyruvate kinase gene sequence. Strain groupings are based on the recent Zheng et al., *Lactobacillus* reclassification [25] and colored as follows, *Lentilactobacillus* purple, *Levilactobacillus* green, *Secundilactobacillus* blue, and *Lactobacillus* yellow. Node colors designate lifestyle as described by Duar et al. [26]. Note: Branches shorter than 0.0127 are shown as having length of 0.127

The nucleotide sequences of *lba0046*, *lba0864*, *lba1426*, and *lba1539* were searched against a previously curated database of 170 *Lactobacillus* genomes updated to incorporate the 25 recently classified genera emended within the genus [25]. Orthologs with > 40% nucleotide sequence identity were mapped to a phylogenetic tree based on the pyruvate kinase (Pyk) enzyme sequence (Fig. 2). The strains used to generate this figure are further detailed in Supplementary Table 1. All identified genes had comparable sizes to their query and consistently exhibited low GC content (< 40%). Several genomes possessed more than one copy of a gene, most often *lba0046*. Figure 2 only depicts S-layer forming strains as none of the investigated genes were present in non-S-layer-formers. Although strains tended to cluster by lifestyle and genera, a clear divide emerged based on the mapped SLAPs (Fig. 2). While only *lba0864* was detected in insect-adapted species, vertebrate adapted species possessed at least two SLAPs in every strain, and several contained all four, including *L. acidophilus*, *L. crispatus*, *L. gallinarum*, *L. kitasatonis*, and *L. ultunensis*. These results are in stark contrast to free-living strains, which encompass three recently identified genera (*Lentilactobacillus*, *Levilactobacillus*, and *Secundilactobacillus*), and were completely devoid of any target SLAPs.

### Chromosomal gene deletion and growth curve analysis

A pORI-based *upp* counterselective gene replacement system [27] was used to generate four separate in-frame deletions within the *L. acidophilus* NCFM chromosome. The mutant strains are listed in Fig. 1 and detailed in Table 1. Deletions were detected by PCR (Supplementary Fig. 1) and sequenced to confirm the absence of both the target SLAP and any unintended mutations within the flanking regions. All SLAP deletion strains were subjected to growth curve analyses. The OD_600_ of the parent and mutant strains was measured over the course of 30 h in MRS, as well as MRS containing 2.5% NaCl, 0.2% porcine bile, and 0.5% oxgall. No differences were detected between any of the strains under these conditions (data not shown).

**Table 1:** Bacterial strains and plasmids used in this study

Strain or Plasmid	Genotype or characteristics	Reference
**Strains**		
***Lactobacillus acidophilus***		
NCK56 (NCFM)	Human intestinal isolate	[28]
NCK1909 (*Δupp*)	NCFM with a 315 bp in-frame deletion within the *upp* gene (*lba0770*); background/parent strain for NCFM deletion mutants	[27]
NCK1910	NCK1909 harboring the *repA* helper plasmid pTRK669; host for pORI-based counterselective integration vector	[27]
NCK2439 (Δ*lba0864*)	NCK1909 with a 1425 bp in-frame deletion within the *lba0864* gene	This study
NCK2441 (Δ*lba1426*)	NCK1909 with a 717 bp in-frame deletion within the *lba1426* gene	This study
NCK2530 (Δ*lba0046*)	NCK1909 with a 342 bp in-frame deletion within the *lba0046* gene	This study
NCK2608 (Δ*lba1539*)	NCK1909 with a 498 bp in-frame deletion within the *lba1539*gene	This study
***Escherichia coli***		
EC101	RepA^+^ JM101; Km^r^; *repA* from pWV01 integrated in chromosome; cloning host for pORI-based plasmids	[29]
NCK1391	*E. coli* DH10B harboring pTRK669; cloning host for pORI-based plasmids	[27]
NCK1911	EC101 host harboring pTRK935 integration vector	[27]
NCK2438	EC101 host harboring pTRK1118 recombinant plasmid	This study
NCK2440	EC101 host harboring pTRK1119 recombinant plasmid	This study
NCK2529	EC101 host harboring pTRK1126 recombinant plasmid	This study
NCK2607	DH10B host harboring pTRK1170 recombinant plasmid	This study
**Plasmids**		
pTRK669	Ori (pWV01), Cm^r^, RepA^+^, thermosensitive	[30]
pTRK935	pORI *upp*-based counterselective integration vector, Em^r^	[27]
pTRK1118	pTRK935 harboring a mutated copy of *lba0864* gene cloned into HindIII/SacI site	This study
pTRK1119	pTRK935 harboring a mutated copy of *lba1426* gene cloned into HindIII/SacI site	This study
pTRK1126	pTRK935 harboring a mutated copy of *lba0046* gene cloned into HindIII/SacI site	This study
pTRK1170	pTRK935 harboring a mutated copy of *lba1426* gene cloned into BamHI/SacI site	This study

### Examination of SLAP mutant cellular morphologies

Flow cytometry and scanning electron microscopy (SEM) were used to detect morphological changes in log phase (6 h) and early stationary phase (12 h) SLAP mutants compared to the parent. Results showed relatively little difference between the strains at either time point (Supplementary Fig. 2). Although NCK2608 (Δ*lba1539*) cells appeared slightly longer in stationary phase flow data, this difference was not discernable from SEM images (Supplementary Fig. 2B). Overall, the strains seemed unencumbered, with no obvious deletion-dependent morphological alterations.

### Deletion of SLAP genes reduces the adhesive capacity of *L. acidophilus*

**Fig. 3:**
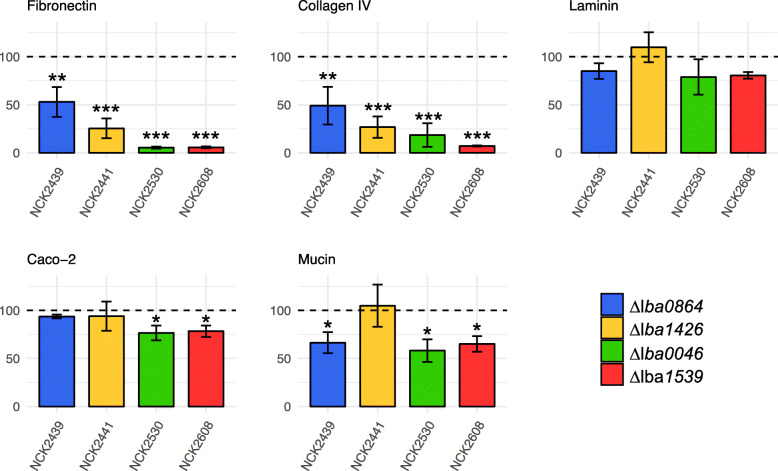
Relative adherence of the SLAP mutants to major extracellular matrices, Caco-2 epithelial cells, and mucin. The *L. acidophilus* NCK1909 parent strain was standardized to 100% (dotted line). The data represent means of independent biological replicates. Error bars are standard error of the means. Asterisks indicate statistical significance calculated using a Student’s t-test (*** *p* < 0.001, ** *p* < 0.01, * *p* < 0.05)

The impact of the SLAP gene deletions on the adhesive capacity of the mutant strains was evaluated using an in vitro adhesion assay. Tested substrates included major ECMs, mucin, and Caco-2 intestinal cells. Assay results were substrate-dependent (Fig. 3). All four mutants demonstrated extensive reductions in adherence to fibronectin and collagen, particularly NCK2608 (Δ*lba1539*) which underwent a > 90% drop relative to the parent strain. Conversely, there were no statistically significant reductions in laminin binding. Despite the relative magnitude of some of the ECM results, Caco-2 adherence deficiencies were only apparent for two of the mutant strains, NCK2530 (Δ*lba0046*) and NCK2608 (Δ*lba1539*), both with relative reductions just over 20%. NCK2441 (Δ*lba1426*) was the only strain to not present some form of mucin-binding deficiency, while the other three mutants demonstrated reductions between 33 and 42%. All strains exhibited similar susceptibility to diluted Triton X-100, eliminating it as a potential source of variability (data not shown).

### SLAP deletion-induced alterations to murine dendritic cell (DC) cytokine profiles

**Fig. 4:**
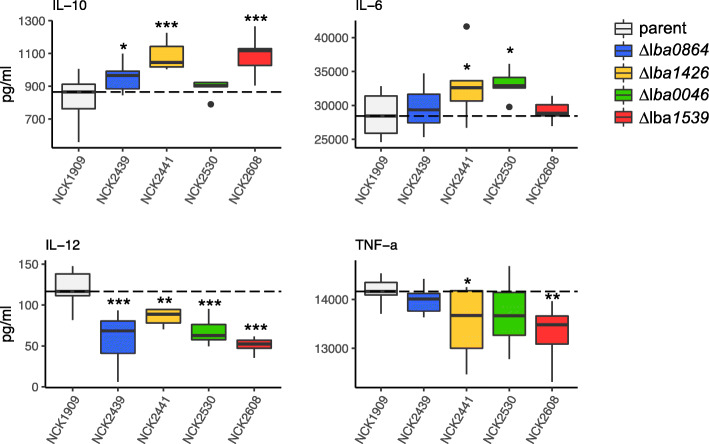
The immunomodulatory profiles of the SLAP mutants compared to the parent strain were evaluated using a murine dendritic cell co-incubation assay. Cytokines IL-10, IL-6, IL-12 and TNF-α, were measured using enzyme-linked immunosorbent assays. Co-incubations were performed in biological triplicate; bars on the box-whisker plots were constructed using block centered data. The dotted line is used to indicate the median of the control strain. Asterisks indicate statistical significance calculated using a Student’s t-test (*** p < 0.001, ** p < 0.01, * p < 0.05)

A bacterial/DC co-incubation assay was used to assess the immunomodulatory potential of LBA0046, LBA0864, LBA1426, and LBA1539. Mutant *L. acidophilus* strains and the parent were exposed to murine DCs for 24 h followed by the measurement of relevant cytokines. To minimize random error, biological replicates were treated as a blocking factor. The block centered data is plotted in Fig. 4. Results indicate that the absence of the four genes produced notably different, predominantly anti-inflammatory, cytokine profiles in comparison to the parent strain. Anti-inflammatory molecule IL-10 was significantly induced by three of the four strains, particularly NCK2441 (Δ*lba1426*) and NCK 2608 (Δ*lba1539*). Likewise, inflammatory molecule IL-12 was repressed by all four strains, while NCK2441 (Δ*lba1426*) and NCK2608 (Δ*lba1539*) reduced TNF-α production. Interestingly, two of the strains, NCK2441 (Δ*lba1426*) and NCK2530 (Δ*lba0046*), slightly but significantly induced pro-inflammatory molecule IL-6.

### Transcriptomic analysis of SLAP deletion mutants

**Fig. 5:**
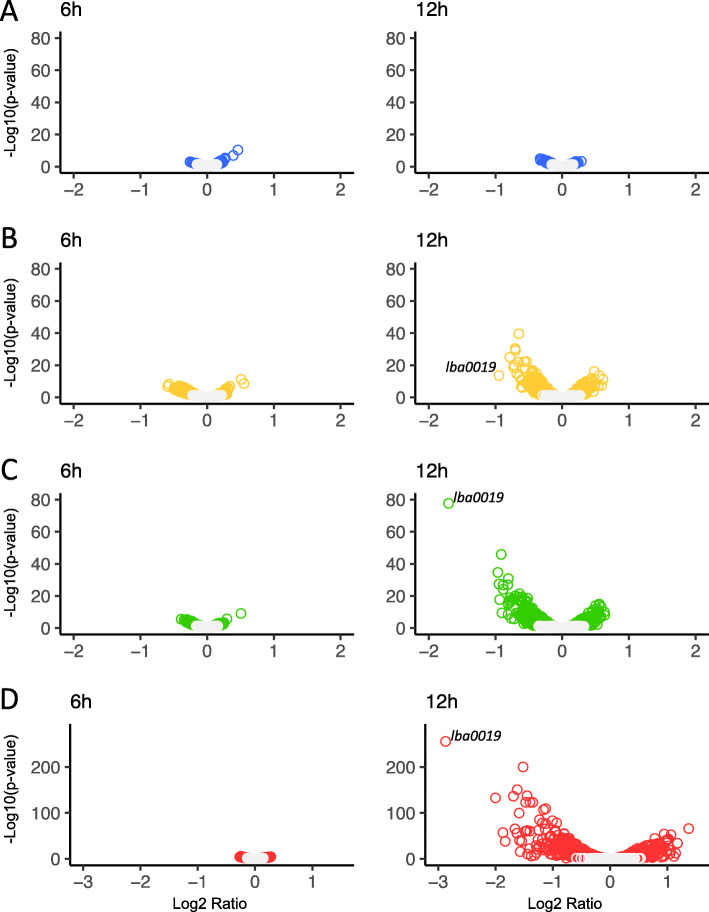
Transcriptome profiles of SLAP mutants compared to the parent strain. The transcriptomes of NCK2439 (Δ*lba0864*, A), NCK2441 (Δ*lba1426*, B), NCK2530 (Δ*lba0046*, C) and NCK2608 (Δ*lba1539*, D) relative to the parent strain (NCK1909). Strains were grown in MRS broth for 6 h (left) and 12 h (right). Volcano plots depict Log2 fold change in expression plotted against significance. The gray circles represent insignificant values, while colored circles are indicative of a *p*-value < 0.05

The global transcriptomes of the four SLAP mutant strains relative to the NCK1909 parent were examined in log (6 h) and early stationary (12 h) growth phases. For both conditions, the Log2 ratio was plotted against statistical significance (Fig. 5). The gray circles represent insignificant values, while colored circles are indicative of a *p*-value < 0.05. At 6 h, all four mutants demonstrated relatively little difference in expression, but by 12 h only the transcriptome of NCK2439 (Δ*lba0864*) still resembled that of the parent strain (Fig. 5A). Although the transcriptomes of the other three mutants appear quite dissimilar, only NCK2530 (Δ*lba0046*) and NCK2608 (Δ*lba1539*) had differentially expressed genes with Log2 ratios > 1 (Fig. 5B-D). Indeed, NCK2608 (Δ*lba1539*) was most influenced by growth phase (Fig. 5D) with 65 differentially expressed genes (Log2 fold change > 1, *p*-value < 0.0001) which are listed in Table 3 along with their corresponding COGs. Of those genes, 56 were repressed, including six related to cell wall/membrane structure and biogenesis, and 11 predicted to play roles in nucleotide/amino acid transport and metabolism. The vast majority of the remaining genes are poorly characterized or possess unknown functions. Upregulated genes were fewer and encompassed transcription anti-terminator *licT*, transcriptional regulator *lysR*, a multiple sugar metabolism (*msm*) operon regulator, bifunctional protein *pyrR*, phosphoribosylformylglycinamidine synthase subunit *purS* and four hypothetical proteins. Remarkably, the most notably downregulated gene, *lba0019*, was the same for NCK2441, NCK2530, and NCK2608 with Log2 ratios of − 0.9, − 1.7, and − 2.9, respectively. This gene encodes an uncharacterized protein predicted to contain an alpha/beta hydrolase fold and exhibited 50% amino acid identity to several *Lactobacillus* esterases. It is located in an operon with putative membrane protein, *lba0018*.

## Discussion

S-layer associated proteins (SLAPs) of *L. acidophilus* have been linked to a broad range of functions including some of the critical roles once believed to be the sole responsibility of the S-layer [7, 18]. In the present study, four putative uncharacterized SLAPs, LBA0046, LBA0864, LBA1426, and LBA1539, were selected for functional analysis. The only consistent feature among the target loci was the possession of an N-terminal signal peptide, which denotes transfer across the cytoplasmic membrane via the Sec translocase system, and confirms their extracellular localization [31]. The nucleotide sequences encoding these proteins were searched for in a curated database of 170 genomes [32]. Orthologs were only present in S-layer-forming strains, which is in agreement with previous SLAP studies [19, 32]. Although *Lactobacillus* species tend to cluster based on lifestyle [26], mapping of SLAP presence added an additional level of granularity. Examined SLAPs were confined to host-adapted *Lactobacillus* species, while *lba0046*, *lba1426*, and *lba1539* were unique to vertebrates. Interestingly, no SLAPs were detected in the free-living strains, which represented three newly identified genera (*Lentilactobacillus*, *Levilactobacillus*, and *Secundilactobacillus*). Likewise, their low GC content is consistent with the genomes of host-adapted species which have lost DNA repair genes and undergone subsequent mutational bias toward A and T [26]. The absence of these proteins from free-living *Lactobacillus* species suggests that SLAPs evolved expressly for the purpose of host adaption.

The genes encoding the four SLAPs of interest were deleted from the *L. acidophilus* NCFM chromosome via a pORI-based *upp* counterselective gene replacement system [27]. Our previous proteomic analysis of the *L. acidophilus* SLAP profile revealed the upregulation of LBA0046, LBA1426, and LBA1539 during the transition from log to stationary growth phase; in fact, LBA0046 and LBA1539 were among the most induced proteins within the dataset [24]. Expression analyses for Δ*lba0046*, Δ*lba1426,* Δ*lba1539* at 6 h and 12 h supported these findings by only demonstrating differential gene expression during stationary growth phase. These results were most apparent for Δ*lba1539* which underwent considerable gene repression, most notably for cell wall biogenesis and structural genes, as well as an aggregation promoting factor (*apf*) protein previously shown to contribute to the survival of *L. acidophilus* during transit through the digestive tract and predicted to mediate host interactions [33]. Surprisingly, in comparison to the parent, the most significantly downregulated gene, *lba0019*, was the same for all three strains. This gene likely encodes an esterase, and is co-transcribed with a putative membrane protein. Esterases are a type of hydrolase enzyme which catalyze the cleavage and formation of ester bonds and have wide substrate specificities [34]. Although we were unable to find more information about the specific role of this protein, its orthologs appear to be confined to host-adapted S-layer-forming lactobacilli.

Examination of mutant cellular morphologies via flow cytometry and SEM revealed no obvious differences in comparison to the parent strain. This data, coupled with unaltered growth, even under stressful conditions, suggests that the mutant cell surfaces remained intact. Prior deletion studies of *L. acidophilus* SLAPs yielded similar results [18, 22], and imply a more direct mechanism for the observed phenotypes, as opposed to SLAPs involved in cell wall maintenance, such as CdpA and IgdA, whose deletion phenotypes were presumed to be pleiotropic responses resulting from disordered cell surfaces [20, 32]. This inferred directness is most apparent for LBA0864, whose deletion had almost no effect on the transcriptome, yet its corresponding mutant strain exhibited significant adherence reductions and immunomodulatory shifts in comparison to the parent. Additionally, LBA0864 was the only examined protein to possess a GW anchor. The GW domain, termed for a conserved Gly-Trp (GW) dipeptide, constitutes a motif for cell-surface anchoring in *Listeria* and other Gram-positive bacteria [35]. Within *Listeria*, this domain enables the protein LnlB to re-associate to the bacterial cell, even when added from extracellular medium, and is thought to play a role in cell invasion [36]. These characteristics support the assumption that LBA0864 has a surface localized function likely related to host interaction.

In general, surface proteins of *L. acidophilus* NCFM are important mediators of adhesion to intestinal epithelial cells, mucus, and extracellular matrices [7, 22, 37, 38]. Among the remaining three mutant strains tested, binding deficiencies were found to be substrate-specific, again consistent with a more direct mechanism of action. In a previous study which mutated the mucus-binding (Mub), fibronectin-binding (FbpA), and SlpA proteins of *L. acidophilus*, resultant strains established that multiple proteins individually contribute to the organism’s ability to adhere to intestinal cells in vitro [7]. In fact, the authors argue that the severe binding deficiency of the *slpA* mutant was likely due to the loss of multiple proteins that may have been embedded within the S-layer. Our results support this claim by demonstrating the influence of several uncharacterized surface proteins on adherence to not only Caco-2 intestinal cells, but also specific extracellular matrices and mucin. Bacteria that are only able to adhere to mucus, but unable to associate with the epithelium, may be washed away with degraded mucins, thus adhesion to extracellular matrices is critical to the realization of certain probiotic attributes [2]. Despite the severe reductions in fibronectin and collagen adhesion, examined SLAPs were all devoid of signature binding domains. Nonetheless, when compared to an *L. acidophilus* mutant with a deleted Type-III fibronectin binding protein [22], our Δ*lba0046* and Δ*lba1539* strains demonstrated substantially more dramatic relative reductions in fibronectin adhesion. Although these four SLAPs do not possess any sequence homology, it is possible that they contain novel domains which contribute to the adhesive capacity of *L. acidophilus*.

For host-adapted bacteria, extracellular features are critical for not only adhesion, but also immunomodulation. The capacity of lactobacilli to variably induce IL-12 and TNF- α, and to a lesser extent, IL-6 and IL-10, may determine which immune response is favored [9]. In comparison to other lactobacilli, *L. acidophilus* NCFM tends to exhibit a slight proinflammatory profile with a very low IL-10/IL-12 ratio [39] that has been directly linked to S-layer presence [17]. The deletion mutants in this study were associated with less inflammatory phenotypes based on either the significant induction of IL-10, reduction of IL-12, or both. This was most apparent with NCK2608 (Δ*lba1539*), which also triggered a significant drop in TNF-α. Unexpected was the slight upregulation of pro-inflammatory molecule IL-6 by Δ*lba0046* and Δ*lba1426*, results which could be attributed to low-level induction of auxiliary surface molecules, though statistical significance was much lower in comparison to other evaluated cytokines. Overall, our results suggest that SLAPs contribute to the slight proinflammatory profile of *L. acidophilus*, a characteristic that was once attributed to the S-layer.

## Conclusions

In summary, the data presented in this study collectively links several previously uncharacterized extracellular proteins to underlying mechanisms contributing to *L. acidophilus* probiotic activity. Initial in silico findings revealed high conservation of these genes among host-adapted S-layer forming lactobacilli and suggest a probable host-related function. Complementary in vitro assay results supported this claim, particularly in regards to host adhesion and immune stimulation, both critical for probiotic functionality. Mutant transcriptome profiles, although not exceptionally divergent from the parent, did highlight a similarly repressed putative esterase whose future functional characterization may indirectly broaden our understanding of the SLAP gene subset. Overall, deciphering the complex adaptative features that promote both survival and persistence of probiotics in the host intestines is essential for elucidating and augmenting their health benefits. Our results demonstrate both the importance of SLAPs in host adaptation and add to the ever-expanding catalog of probiotic-relevant molecules.

## Methods

### Sequence analysis and phylogenetic tree mapping

Genetic context was examined by extracting previously annotated sequences encoding LBA0046, LBA0864, LBA1426, LBA1539 and flanking regions from the *L. acidophilus* NCFM genome (NC_006814) using Geneious software [40]. Protein domains were detected and assigned using UniProt and InterPro databases [41, 42]. Phylogenetic mapping was conducted to gain insight into the presence of these loci within a database of 170 genomes [5] updated to take into account the recently published *Lactobacillus* genus reclassifications [25]. Orthologs were identified and extracted via Geneious annotation and extraction workflows [40], then imported into CLC Genomics Workbench (Qiagen). S-layer presence was determined using the UniProt annotation tool suite to search for proteomes present within the UniProt database, as previously described [32]. A phylogenetic tree containing only S-layer-forming bacteria was constructed using a published method based on the nucleotide sequence of the Pyk enzyme [5, 43]. SLAP orthologs and Duar et al. [26] species lifestyles were mapped to the tree using the CLC Genomics metadata feature, however *L. acidophilus* NCFM was re-classified as vertebrate-adapted [32].

### Bacterial strains and growth conditions

The bacteria and plasmids used in this study are listed in Table 1, while Tables 2 and 3 details the PCR primers required for generating the mutant strains. *Escherichia coli* EC101 acted as a host for cloning the deletion constructs for all genes except *lba1539*, for which NCK1391 (*E. coli* DH10B harboring pTRK669) was substituted. *E. coli* EC101 was propagated in brain heart infusion (BHI) broth (Difco Laboratories, Detroit, MI) with aeration at 37 °C in the presence of 40 μg/ml kanamycin (Sigma-Aldrich, St. Louis, MO, USA), while NCK1391 was grown in Luria-Bertani (LB) broth (Difco) with aeration at 32 °C in the presence of 15 μg/ml chloramphenicol. Recombinant *E. coli* cells containing pTRK935-based plasmids were selected using 150 μg/ml erythromycin. Propagation and selection of *Lactobacillus* strains was performed as previously described [32]. For growth curve analyses, overnight bacterial cultures were used to inoculate 96-well microplates (Corning Costar, Corning, NY) containing MRS broth or MRS broth supplemented with 2.5% (w/v) NaCl (Fisher Scientific, Hampton, NH, USA), 0.2% (w/v) porcine bile (Sigma) or 0.5% (w/v) oxgall (Difco). Plates were sealed with clear adhesive film then incubated at 37 °C in a Fluostar Optima microplate reader (BMG Labtech, Cary, NC). The OD_600_ was measured every hour for 30 h.

**Table 2:** PCR primers used in this study

Primer Name	Sequence*
**Construction of deletion mutants**	
0046HindIIIF	GATCTAAAGCTTGCTCAACATTATTAACGGTTC
0046R	TTCTAACATAATGAATACCTCGTA
0046Soe	AGGTATTCATTATGTTAGAAGACTAATCTAGATCAAGATTCATCA
0046SacIR	GATCTAGAGCTCCATACTACTTCTGCGTCTTC
0864HindIIIF	GATCTAAAGCTTCTGCTGATATTGATGCAGTGAGTGG
0864R	CGCACCGGCAATAACTATTCCCTTAAT
0864Soe	AAGGGAATAGTTATTGCCGGTGCGCGTGCAGAATTAACTCAAGGTCGC
0864SacIR	GATCTAGAGCTCCGTGCACTTGACACAGATCCTG
1426HindIIIF	GATCTAAAGCTTTATAGATTAATTGACTGCAGCC
1426R	CGCTGCCATTGAAGTAATTA
1426Soe	TAATTACTTCAATGGCAGCGAACTAATCTATTAATGAAGAAACTCGT
1426SacIR	GATCTAGAGCTCCTTATCGTTCATGCCAAGAA
1539BamHIF	GATCTAGGATCCATCACTTGATCGATCATCTG
1539R	CTTCATCTGAATATCTCCTCT
1539Soe	AGAGGAGATATTCAGATGAAGTTGATAAAATAATCTACTACTTTGTGA
1539SacIR	GATCTAGAGCTCCTCTTAGGTGCAAGCATTAA
**PCR analysis and DNA sequencing of deletion targets**	
0046up	CTATCTGTATGATGCTTCCAC
0046dw	GTACCTCAATCTGTTGTAATCTC
0864up	ACAAGCTAGAGGTATGGCTGG
0864dw	CCACATGAATGGCGTATGGC
1426up	AAGCCGTTGTATTGAATGATGGTAG
1426dw	CGCGAATCATCAATTCACGGTA
1539up	CAGGATAGGGATGCACATGC
1539dw	CGACGTTGACGTGTTACTGT

*The primers, the 5′-to-3′ sequences are given and restriction enzyme sites are underlined

**Table 3:** NCK2608 (Δ*lba1539*) differentially expressed genes in stationary growth phase

Locus ID	Name	Ratio	COG*
**CELLULAR PROCESSES AND SIGNALING**			
LBA0833	ftsW	−1.06	D
LBA1883	NLP-P60 secreted protein	−2.00	M
LBA1140	lysin	−1.58	M
LBA0520	galactosyltransferase	−1.48	M
LBA1743	cell wall-associated hydrolase	−1.16	M
LBA1744	glycosidase	−1.13	M
LBA1736	epsB	−1.09	M
LBA0165	pepO	−1.40	O
LBA1901	thioredoxin	−1.38	O
LBA1208	msrA	−1.25	O
LBA1107	glutathione reductase	−1.13	O
LBA0096	htpX	−1.12	O
LBA1659	response regulator	−1.63	T
LBA0544	transcriptional regulator	−1.45	T
LBA1132	ABC transporter component	−1.18	V
LBA0560	ABC transporter component	−1.07	V
LBA1680	ABC transporter component	−1.01	V
**INFORMATION STORAGE AND PROCESSING**			
LBA1519	pheS	−1.03	J
LBA1899	transcriptional regulator	−1.44	K
LBA0835	hypothetical protein	−1.29	L
LBA0797	radC	−1.04	L
LBA0545	hypothetical protein	−1.00	L
**METABOLISM**			
LBA1109	hypothetical protein	−1.47	C
LBA1220	pyridine mercuric reductase	−1.45	C
LBA0538	Na + −H + -exchanging protein	−1.09	C
LBA1896	asnA	−1.54	E
LBA1961	oppA	−1.28	E
LBA1177	iron-sulfur cofactor synthesis	−1.26	E
LBA1045	ABC transporter component	−1.15	E
LBA1292	aa transporter	−1.14	E
LBA1658	prolyl aminopeptidase	−1.04	E
LBA0240	xanthine phosphoribosyltransferase	−1.40	F
LBA0041	rtpR	−1.35	F
LBA1631	deoxyribosyltransferase	−1.24	F
LBA0591	iunH	−1.18	F
LBA0131	ribose-p pyrokinase	−1.06	F
LBA0836	coaD	−1.05	H
LBA0542	heavy-metal-transporting ATPase	−1.87	P
LBA1771	ABC transporter component	−1.69	P
LBA0541	cadA	−1.47	P
LBA0200	oppB	−1.06	P
**POORLY CHARACTERIZED**			
LBA0019	hypothetical protein	−2.87	S
LBA0543	hypothetical protein	−1.66	S
LBA0493	aggregation promoting protein	−1.62	S
LBA0834	hypothetical protein	−1.57	S
LBA1769	hypothetical protein	−1.52	S
LBA1943	lipoprotein	−1.40	S
LBA1850	lysM	−1.23	S
LBA0387	hypothetical protein	−1.18	S
LBA0208	hypothetical protein	−1.14	S
LBA1010	hypothetical protein	−1.03	S
LBA1738	hflX	−1.01	S
LBA0018	membrane protein	−1.83	None
LBA1108	hypothetical protein	−1.59	None
LBA0017	general stress response	−1.32	None
LBA1221	hypothetical protein	−1.29	None
LBA0616	hypothetical protein	1.01	M
LBA0724	licT	1.37	K
LBA1410	lysR	1.18	K
LBA1443	msm operon regulator	1.06	K
LBA0563	pyrR	1.14	F
LBA1558	purS	1.11	F
LBA0644	hypothetical protein	1.06	Q
LBA0402	hypothetical protein	1.02	None
LBA1889	hypothetical protein	1.05	None

*Clusters of Orthologous Groups (COG) were assigned to significant genes using the EggNOG Database. The categories are as follows: C, Energy production and conversion; E, amino acid transport and metabolism; F, nucleotide transport and metabolism; G, carbohydrate transport and metabolism; H, coenzyme transport and metabolism; I, lipid transport and metabolism; J, translation, ribosomal structure and biogenesis; K, transcription; L, replication, recombination and repair; M, cell wall/membrane/envelope biogenesis; O, post-translational modification, protein turnover, and chaperones; P, inorganic ion transport and metabolism; Q, secondary metabolites biosynthesis, transport, and catabolism; R, general function prediction only; S, function unknown; T, signal transduction mechanisms; V, defense mechanisms

### Chromosomal deletion of SLAP genes targets

DNA manipulations and transformation was performed as previously described [32]. The SLAPs encoded by *lba0046*, *lba0864*, *lba1426*, and *lba1539* were deleted from the *L. acidophilus* NCFM chromosome via a pORI-based *upp* counterselective gene replacement system [27]. Briefly, in-frame deletions were created by PCR amplifying sequences upstream and downstream of the deletion targets (Table 2). Subsequent purified products were joined by splicing using overlap extension PCR (SOE-PCR), then amplified and cloned into the pTRK935 integration vector. Recombinant plasmids were transformed into *E. coli* and eventually electroporated into *L. acidophilus* NCK1910 (Table 1). Recovery of single- and double-crossover recombinants was performed as previously described [27]. Gene deletions were confirmed by sequencing the entirety of both flanking regions. For all subsequent phenotypic assays, NCK1909 (Δ*upp*) served as the control strain (Table 1).

### Examination of mutant cellular morphologies

Flow cytometry was used to examine changes to cell size and granularity resulting from SLAP gene deletions. Mutant and parent strains were grown to log (6 h) and early stationary (12 h) growth phases in MRS broth. Cells were centrifuged at 3220×*g* for 10 min, then washed and resuspended in phosphate buffered saline (PBS, pH 7.4, Thermo Fisher Scientific, Waltham, MA, USA). Data was acquired using a CytoFLEX Flow Cytometer instrument (Beckman Coulter, Brea, CA, USA) and analyzed with CytExpert software (Beckman Coulter). Changes in morphology were also visualized with SEM. Log (6 h) and early stationary phase (12 h) cells, cultured in MRS broth, were fixed in a solution of 3% glutaraldehyde in 0.1 M sodium cacodylate (pH 5.5) and stored at 4 °C. Fixed cells were processed by the CALS Center for Electron Microscopy (CEM) at North Carolina State University. Images were acquired with a JEOL JEM-5900LV SEM (JEOL USA, Peabody, MA) at 15 kV.

### Adhesion to mucin, extracellular matrices (ECMs), and Caco-2 intestinal cells

Binding assays were performed as previously described [32]. Adhesion substrates consisted of Mucin (Type III from porcine stomach, Sigma), fibronectin (from human plasma, Sigma), collagen (type IV from human cell culture, Sigma), and laminin (from Engelbreth-Holm-Swarm murine sarcoma/basement membrane; Sigma), as well as Caco-2 intestinal cells purchased from the American Type Culture Collection. Relative adherence percentages were calculated by standardizing the parent strain adherence to 100%. Mucin and ECM adhesion assays were performed in biological triplicate with four technical replicates, while Caco-2 adhesion assays were performed a minimum of three times with two technical replicates. Statistical significance was determined using a Student’s t-test.

### Bacteria/DC co-incubation and cytokine quantification

Bone marrow-derived C57BL/6 murine immature dendritic cells (DCs) were purchased from Astarte-Biologics (Bothell, WA) and preserved in liquid nitrogen. Bacterial co-incubation assays were performed as previously described [32]. Cytokine measurements for tumor necrosis factor α (TNF-α) and interleukins IL-6, IL-10 and IL-12 were obtained using Single-Analyte ELISArray kits (Qiagen) as per manufacturer’s instructions. Assays were performed in biological triplicate with two technical replicates. To reduce random error, replicates were treated as a blocking factor. Significance of block centered data was analyzed using a Student’s t-test.

### RNA extraction, sequencing, and transcriptional analysis

Total RNA was isolated from the *L. acidophilus* parent strain and SLAP-deficient mutants propagated in MRS broth for 6 and 12 h. Cells were grown statically under ambient atmospheric conditions, pelleted by centrifugation (3220×g, 5 min, RT), then flash frozen and stored at − 80 °C. RNA isolation and sequencing was performed as previously described by Klotz et al., [32]. Geneious software [40] was used to filter and map reads to the *L. acidophilus* NCFM reference genome and differential expression levels were calculated using the DESeq2 package [28]. Clusters of Orthologous Groups (COGs) were assigned using EggNOG 5.0 [44]. Transcriptomic datasets generated in this study are available in the National Center for Biotechnology database under BioProject ID PRJNA576881.

## Supplementary information

**Additional file 1 Supplementary Fig. 1.** SLAP deletions visualized using PCR. Confirmation of the S-layer associated protein gene deletions from the *L. acidophilus* NCFM chromosome using primers that flanked the deletion region. P, NCK1909 parent strain, Δ, SLAP deletion strain.

**Additional file 2 Supplementary Fig. 2**. Cellular morphologies of the parent strain and SLAP mutants. Cellular morphologies of the parent strain and SLAP mutants in logarithmic (6 h, A) and early stationary (12 h, B) growth phase visualized using flow cytometry and scanning electron microscopy.

**Additional file 3 Supplementary Table 1.** List of strains used, Zheng et al., reclassification, lifestyles, and isolation sources.

## Data Availability

The datasets generated and/or analyzed during the current study are available at the National Center for Biotechnology database under BioProject ID PRJNA576881 (https://www.ncbi.nlm.nih.gov/bioproject/?term=PRJNA576881).

## Abbreviations

GOG: Clusters of Orthologous Groups
DC: Dendritic cell
ECM: Extracellular matrix
LAB: Lactic acid bacteria
SEM: Scanning electron microscopy
S-layer: Surface-layer
SLP: S-layer protein
SLAP: S-layer associated protein
