# Supplementary figures and images for: S-layer associated proteins contribute to the adhesive and immunomodulatory properties of Lactobacillus acidophilus NCFM
Source: BMC Microbiol. 2020 Aug 12;20:248. doi: 10.1186/s12866-020-01908-2 (PMC7425073; doi:10.1186/s12866-020-01908-2)

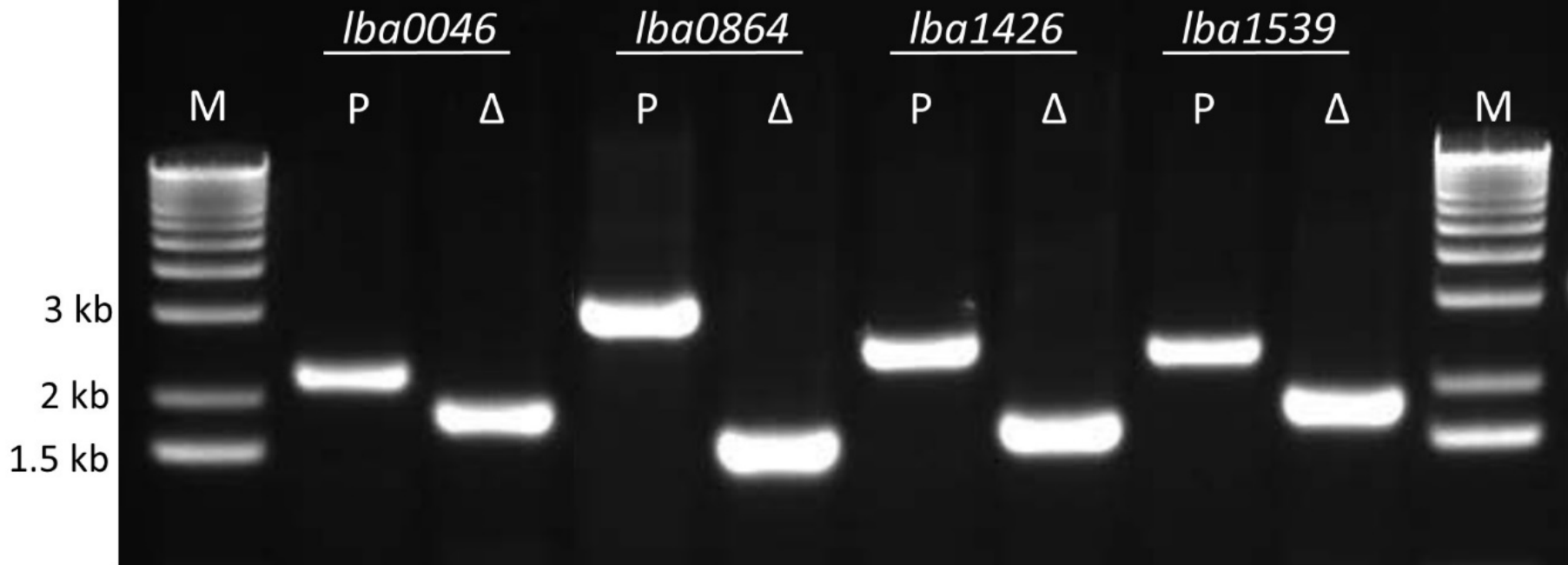

Supplement: Supplementary file 1 — Additional file 1 Supplementary Fig. 1. SLAP deletions visualized using PCR. Confirmation of the S-layer associated protein gene deletions from the L. acidophilus NCFM chromosome using primers that flanked the deletion region. P, NCK1909 parent strain, Δ, SLAP deletion strain. [file 12866_2020_1908_MOESM1_ESM.pdf]

**A**NCK1909  
parent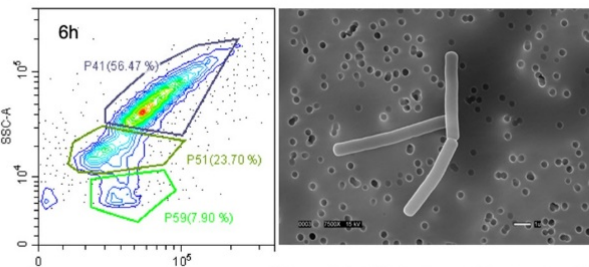NCK2439  
 $\Delta$ lba0864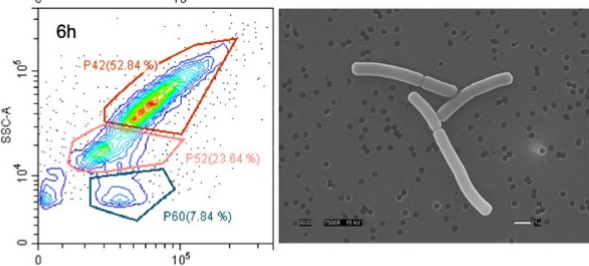NCK2441  
 $\Delta$ lba1426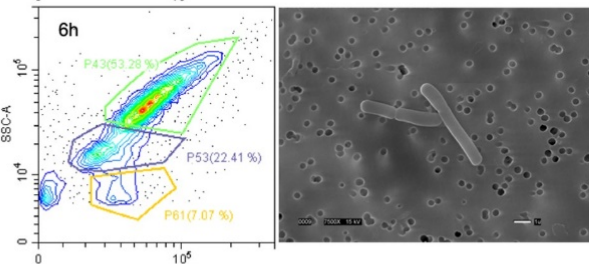NCK2530  
 $\Delta$ lba0046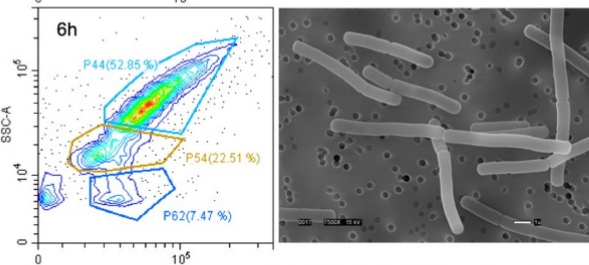NCK2608  
 $\Delta$ lba2608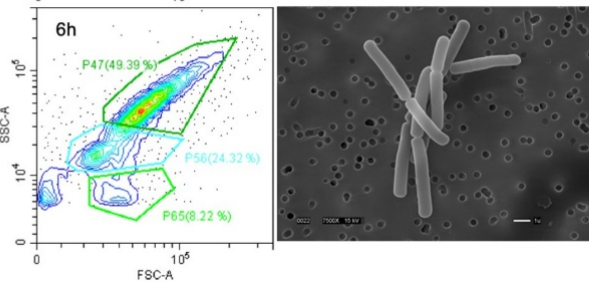**B**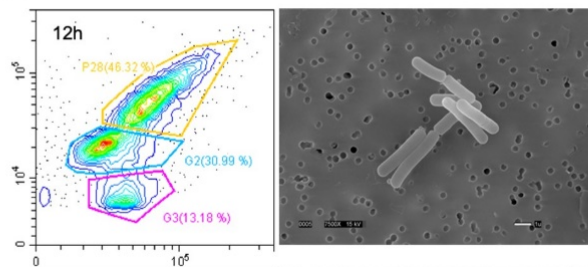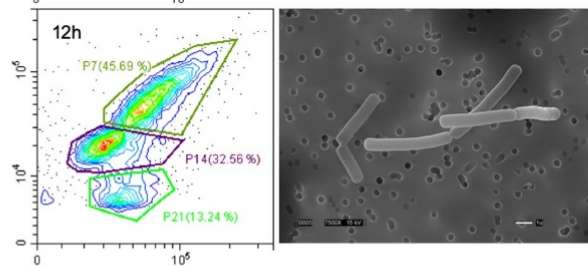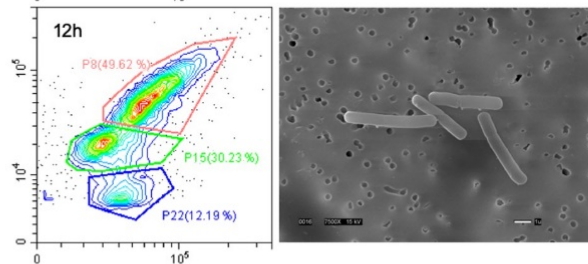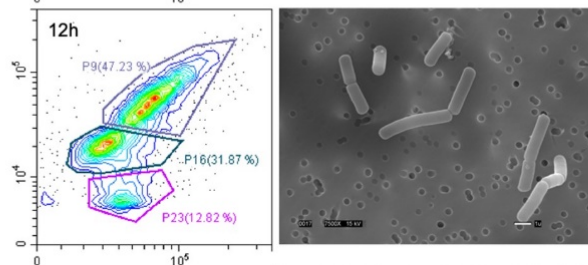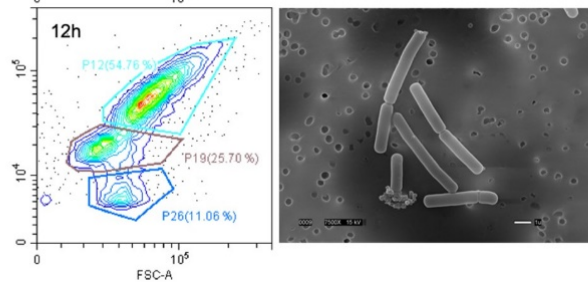

Supplement: Supplementary file 2 — Additional file 2 Supplementary Fig. 2. Cellular morphologies of the parent strain and SLAP mutants. Cellular morphologies of the parent strain and SLAP mutants in logarithmic (6 h, A) and early stationary (12 h, B) growth phase visualized using flow cytometry and scanning electron microscopy. [file 12866_2020_1908_MOESM2_ESM.pdf]
