# Supplementary material for: S-layer associated proteins contribute to the adhesive and immunomodulatory properties of Lactobacillus acidophilus NCFM
Source: BMC Microbiol. 2020 Aug 12;20:248. doi: 10.1186/s12866-020-01908-2 (PMC7425073; doi:10.1186/s12866-020-01908-2)
Supplement: Supplementary file 3 — Additional file 3 Supplementary Table 1. List of strains used, Zheng et al., reclassification, lifestyles, and isolation sources. [file 12866_2020_1908_MOESM3_ESM.docx]

| **Supplementary Table 1 \|** List of strains used, Zheng *et al*., reclassification, lifestyles, and isolation sources. | | | |
| --- | --- | --- | --- |
| ***Lactobacillus* strain** | **Zheng *et al*., reclassification** | **Metadata lifestyle** | **Isolation source** |
| *L. acetotolerans* NBRC 13120 |  |  | sake |
| *L. acidophilus* NCFM |  | Vertebrate-adapted | human gastrointestinal tract |
| *L. amylolyticus* DSM 11664 |  | Vertebrate-adapted | acidified beer wort |
| *L. amylovorus* 30SC |  | Vertebrate-adapted | porcine ileum |
| *L. apis* Hma11 |  | Insect-adapted | honey stomach of honey bee |
| *L. bombicola* R-53102 |  | Insect-adapted | bumblebee gut (Bombus lapidarius) |
| *L. brevis* ATCC 367 | *Levilactobacillus brevis* | Free-living | silage |
| *L. buchneri* CD034 | *Lentilactobacillus buchneri* | Free-living | grass silage |
| *L. collinoides* DSM 20515 | *Secundilactobacillus collinoides* | Free-living | fermenting apple juice |
| *L. crispatus* ST1 |  | Vertebrate-adapted | chicken crop |
| *L. diolivorans* DSM 14421 | *Lentilactobacillus diolivorans* | Free-living | maize silage |
| *L. farraginis* DSM 18382 | *Lentilactobacillus farraginis* | Free-living | composting material of distilled shochu residue |
| *L. gallinarum* HFD4 |  | Vertebrate-adapted | chicken crop |
| *L. gigeriorum* DSM 23908 |  | Vertebrate-adapted | chicken crop |
| *L. hamsteri* DSM 5661 |  | Vertebrate-adapted | feces of hamster |
| *L. helsingborgensis* Bma5 |  | Insect-adapted | honey stomach of honey bee (Apis mellifera mellifera) |
| *L. helveticus* CNRZ32 |  | Vertebrate-adapted | artisanal starter, Comte´ cheese |
| *L. hilgardii* ATCC 8290 | *Lentilactobacillus hilgardii* | Free-living | wine |
| *L. intestinalis* DSM 6629 |  | Vertebrate-adapted | intestine of rat |
| *L. kalixensis* DSM 16043 |  | Vertebrate-adapted | gastric biopsies, human stomach mucosa |
| *L. kefiranofaciens* ZW3 |  |  | Tibet kefir |
| *L. kefiri* DSM 20587 | *Lentilactobacillus kefiri* | Free-living | kefir grains |
| *L. kimbladii* Hma2 |  | Insect-adapted | honey stomach of honey bee (Apis mellifera) |
| *L. kimchicus* JCM 15530 | *Secundilactobacillus kimchicus* |  | kimchi |
| *L. kisonensis* DSM 19906 | *Lentilactobacillus kisonensis* | Free-living | non-salted pickle solution used in production of sunki |
| *L. kitasatonis* DSM 16761 |  | Vertebrate-adapted | chicken intestine |
| *L. kullabergensis* Biut2 |  | Insect-adapted | honey stomach of honey bee (Apis mellifera mellifera) |
| *L. melliventris* Hma8 |  | Insect-adapted | Apis mellifera Buckfast (honey stomach) |
| *L. odoratitofui* DSM 19909 | *Secundilactobacillus odoratitofui* |  | fermented brine used for stinky tofu production |
| *L. otakiensis* DSM 19908 | *Lentilactobacillus otakiensis* | Free-living | non-salted pickle solution used in production of sunki |
| *L. parabuchneri* FAM21731 | *Lentilactobacillus parabuchneri* | Free-living | Swiss Emmental cheese |
| *L. paracollinoides* strain TMW 1.1994 | *Secundilactobacillus paracollinoides* | Free-living | brewery environment |
| *L. parafarraginis* DSM 18390 | *Lentilactobacillus parafarraginis* | Free-living | composting material of distilled shochun residue |
| *L. parakefiri* DSM 10551 | *Lentilactobacillus parakefiri* | Free-living | kefir grain |
| *L. pasteurii* DSM 23907 |  | Vertebrate-adapted | N/A |
| *L. rapi* DSM 19907 | *Lentilactobacillus rapi* | Free-living | non-salted pickle solution used in production of sunki |
| *L. silagei* JCM 19001 | *Secundilactobacillus silagei* |  | orchardgrass silage |
| *L. similis* DSM 23365 | *Secundilactobacillus similis* | Free-living | fermented cane molasses at alcohol plants |
| *L. sunkii* DSM 19904 | *Lentilactobacillus sunkii* | Free-living | non-salted pickle solution used in production of sunki |
| *L. ultunensis* DSM 16047 |  | Vertebrate-adapted | gastric biopsies, human stomach mucosa |
